# Scrotal calciphylaxis in a fifty-one-year-old man with end-stage renal disease and prior bacteremia

**DOI:** 10.1016/j.eucr.2023.102564

**Published:** 2023-09-19

**Authors:** Piroz Bahar, Jennifer Mancuso, Karthik Ramani, Anastasia Wasylyshyn, George Wasylyshyn, Arvin George

**Affiliations:** a University of Michigan Medical School, USA; b University of Michigan Michigan Medicine, USA; c Johns Hopkins Medicine, USA

## Abstract

Isolated, ischemic scrotal lesions are an uncommon manifestation of systemic calciphylaxis. This highly morbid disease seen in patients with end-stage renal disease and other risk factors for small vessel calcification results in tissue necrosis and ulceration. Scrotal calciphylaxis is uncommon and difficult to diagnose in patients with comorbid conditions that cause isolated genital skin lesions, including prior systemic infections. We describe a 51-year-old male with end-stage renal disease on hemodialysis and recent history of bacteremia who developed isolated penile and scrotal ulceration due to calciphylaxis. The patient died two months after presentation despite multidisciplinary care.

## 1 Introduction

Calciphylaxis is a rare condition that primarily affects patients with end-stage renal disease (ESRD) undergoing hemodialysis and those with other risk factors for small vessel calcification, including calcium-containing phosphate binders, excess vitamin D intake, hyperparathyroidism, hypoalbuminemia, autoimmune conditions, bone-mineral disorders, vitamin K antagonists, and warfarin usage.^1^ The disease results from calcification of the medial layer of arterioles and small arteries. Decreased perfusion in calciphylaxis leads to tissue ischemia, necrosis, and ulceration.^1^ Patients typically present with painful skin lesions, superimposed infections, non-healing wounds, and severe pain, though it is uncommon for the disease to present with isolated scrotal lesions.^2^ We report the case of a patient with rapidly progressing scrotal calciphylaxis with a prior medical history of ESRD with hemodialysis and recent bacteremia.

## 2 Case presentation

**Fig. 1:**
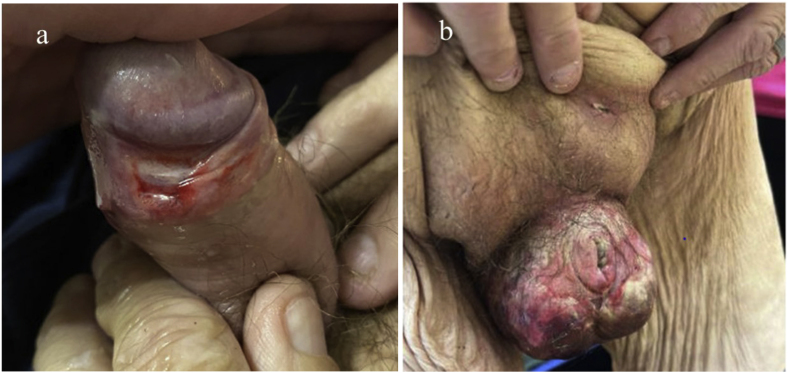
**Clinical Images of the Patient's Penis and Scrotum Prior to Hospitalization.** a: Presentation to primary care physician three weeks prior to presentation. Non-circumcised male with thickening of the preputial foreskin. Dusky erythema of the corona and blanching of the glans penis. The foreskin was retractable with a visible patent urethral meatus and ulcer formation on the glans. b: Presentation to urology as outpatient with edema of the scrotal wall with superficial erosions and white exudate. The foreskin was thickened, phimotic, and unable to be retracted to expose the glans. He was referred to ED for progression in clinical status.

A 51-year-old man presented to outpatient urology with progressive severe genital pain, scrotal ulceration and eschar, phimosis, and urinary retention. His medical history included type 2 diabetes mellitus, stage 5 chronic kidney disease initiated on dialysis three months prior to presentation, peripheral and coronary artery disease, and penile ulceration treated with topical ketoconazole cream and oral valacyclovir three weeks prior to presentation (Fig. 1a). Six months prior to presentation, he had a coronary artery bypass graft complicated by a methicillin-susceptible *Staphylococcus aureus* and *Streptococcus mitis* sternal wound infection, which resulted in bacteremia and required sternal debridement. His physical exam was notable for scrotal duskiness, tenderness, and significant swelling, causing penile phimosis that buried his penis and urethra (Fig. 1b). His condition warranted transfer to the emergency department, where his lab tests were notable for leukocytosis (14.3 K/u), neutrophilia (90.1%), and elevated c-reactive protein (26.7 mg/dL). His ionized calcium, parathyroid hormone, and phosphorous levels were also elevated at 1.36 mmol/L, 319 pg/mL, 8.3 mg/dL, respectively. Urinalysis showed moderate bacteria (culture was positive for Enterococcus faecium in 10,000–100,000 colony-forming units).

Punch biopsy of the scrotum showed epidermal and dermal necrosis with vascular congestion and associated interstitial neutrophilic infiltrate but was negative for microorganisms and calcium. Tissue cultures were positive for *Enterococcus faecium* and *Enterobacter cloacae*. The patient was started on linezolid and meropenem per organism susceptibilities. Fluconazole was continued given concerns for candidal balanitis.

**Fig. 2:**
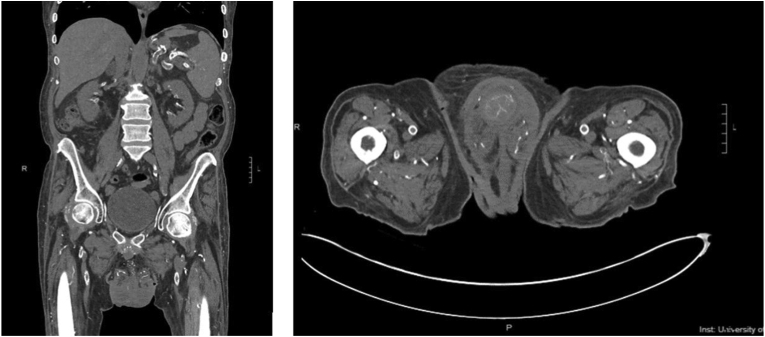
Non-Contrast computed tomography scan of patient's abdomen and pelvis: Significant microvascular calcification within large and small order vessels of the patient's genitals and pelvis, including extensive calcifications of penile vasculature.

**Fig. 3:**
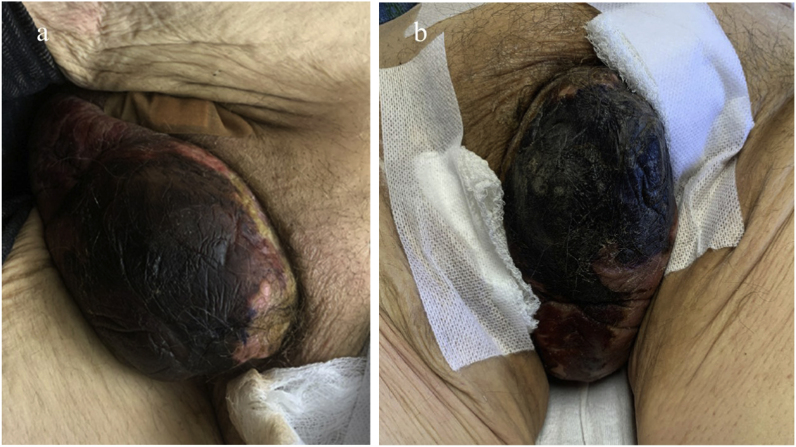
**Clinical Images of the Patient's Penis and Scrotum During Hospitalization.** a: Hospital Day 4. Continued progression of scrotal findings with early onset dry gangrene. The scrotum appears necrotic with formation of a superficial eschar. b: Hospital day 11. Dry gangrene is present with a mature eschar which is woody hard on examination. The foreskin is completely stenosed and no longer visible. There is no evidence or purulent discharge or superficial exudate.

Non-contrast CT imaging of the abdomen and pelvis (Fig. 2) showed prominent portocaval and left para-aortic lymph nodes and extensive atherosclerotic changes seen with vascular calcification of the penis and calcification of the corpus cavernosa fascia. There was no evidence of Fournier's gangrene or the presence of a gas-forming organism. On physical exam four days after admission, the scrotum appeared uniformly necrotic with enlarging eschar (Fig. 3a). There was no evidence of fluctuance or purulent drainage. Calcium supplementation was discontinued, and oral tadalafil and a low phosphate diet were initiated. Inpatient palliative care was consulted to treat severe pain. Surgical debridement of the patient's scrotum was not recommended due to poor blood supply to his genitals and likelihood of creating a non-healing, open wound.

On the 11th day of his hospitalization, the patient's scrotal wound stabilized (Fig. 3b). On day 13, the patient was discharged from the hospital to his home with continued cefepime and linezolid antibiotic treatment. Sodium thiosulfate was initiated at the end of antibiotic therapy. For pain, he was prescribed 4 mg Dilaudid to take prior to hemodialysis with 3 mg as needed, topical nitroglycerin, and morphine. He also received a bilateral genitofemoral nerve block. He developed additional necrotic lesions on his left leg, right thigh, and left hip, as well as pressure ulcerations on his left side. Methadone was initiated in response to the patient's resulting increased pain. As his condition continued to deteriorate, he was enrolled in hospice care and was supported leading up to his death two months after presentation.

## 3 Discussion

Scrotal calciphylaxis is a rare complication of systemic calcific uremic arteriolopathy, a highly morbid disease seen in individuals with end-stage renal disease, co-occurring bone mineral disorders, and chronic inflammatory states.^3^, ^4^, ^5^ The 1-year mortality rate for the condition is greater than 50%.^1^^,^^2^ Patients with calciphylaxis present with a broad range of cutaneous and subcutaneous lesions that look similar to those caused by other diseases, such as Fournier's gangrene, vasculitis, purpura fulminans, oxalate vasculopathy, cholesterol embolism syndrome, and warfarin-induced skin necrosis.^4^^,^^6^

Calciphylaxis treatment is targeted at reversing the pathophysiologic processes that lead to microvessel calcification and tissue ischemia, caring for calciphylaxis related wounds, optimizing dialysis clearance, and addressing pain. In cases where pain does not respond to high-dose opioids, sciatic nerve cryoneurolysis or pudendal nerve blocks can be employed.^7^^,^^8^ Patients with calciphylaxis are instructed to follow a low calcium diet, limit calcium-based phosphate binders, and avoid vitamin D analogs. Kidney transplantation and increased dialysis dosing regimens have also been proposed as treatment options to increase renal calcium clearance.^9^^,^^10^ However, both treatments lack data supporting their effectiveness. Similarly, intravenous or intralesional sodium thiosulfate (STS) is commonly used as a treatment for calciphylaxis because thiosulfate has a vasodilatory effect and creates soluble calcium thiosulfate complexes, which decreases calcium-phosphate precipitation in vascular tissue.^7^^,^^11^ STS can be used alongside other vasodilatory medications, including phosphodiesterase-5 inhibitors, to reverse calciphylaxis related tissue ischemia. Patients with pathologic hyperparathyroidism can consider taking calcimimetic agents, including cinacalcet, taking bisphosphonates, or undergoing a parathyroidectomy.^7,11^ Wound debridement to remove necrotic tissue can be offered to patients with the recognition that patients with calciphylaxis are more likely to develop non-healing wounds and therefore be poor surgical candidates. Finally, hyperbaric oxygen therapy can be offered to patients to increase the oxygenation of necrotic tissue.

It is important to note that while these treatments can be offered to patients, calciphylaxis continues to have a mortality rate between 45 and 80% within one year. Thus, clinicians should be prepared to coordinate with palliative care to engage in end-of-life discussions and treat severe pain caused by the disease.^1^

## 4 Conclusion

This case shows the challenges of diagnosing and treating calciphylaxis given the inconclusive nature of diagnostic tools and lack of evidence-based, effective treatments. This underscores the importance of early clinical suspicion of the disease, even when patient presentation is atypical and diagnostic data is inconclusive.
